# Nutritional composition and bioactivity of germinated Thai indigenous rice extracts: A feasibility study

**DOI:** 10.1371/journal.pone.0237844

**Published:** 2020-08-24

**Authors:** Manat Chaijan, Worawan Panpipat

**Affiliations:** Food Technology and Innovation Research Center of Excellence, Department of Agro-Industry, School of Agricultural Technology, Walailak University, Thasala, Nakhon Si Thammarat, Thailand; Institute for Biological Research "S. Stanković", University of Belgrade, SERBIA

## Abstract

The feasibility of the production of germinated rice extracts using indigenous rice from Southern Thailand, including *Khemtong* (KHT) and *Khai Mod Rin* (KMR) from a single location at the Pak Phanang River Basin in Nakhon Si Thammarat, was investigated. The nutritional composition and bioactivity of the germinated rice extracts from both cultivars were evaluated. Optimum germination time for both rices was 96 h, leading to the highest GABA, thiamine, free amino acid, total sugar, and α-amylase activity (p<0.05). Germinated KHT had a higher α-amylase activity than germinated KMR at all germination times. Mashing at 60°C/pH 5.5 rendered the extract with the highest GABA content (p<0.05) and desirable contents of other nutrients. In comparison with germinated *Sungyod* (local colored rice) and *Jasmine* (commercial Thai rice) extracts, KHT and KMR showed a higher scavenging activity against DPPH^•^, OH^•^, and H_2_O_2_ (p<0.05) with a comparable ABTS^•+^ inhibition. For metal chelation, reducing power and ACE inhibitory activity, the germinated *Sungyod* extract was greater than KHT/KMR. The results demonstrated the potential use of germinated local Thai rice for the production of functional beverages.

## Introduction

Thailand is one of the major Asian countries for rice (*Oryza sativa* L.) production. Different regions in Thailand cultivate different traditional rice varieties. In Southern Thailand, *Khai Mod Rin* (NSRC9500113) (KMR) and *Khemtong* (PTLC9700142) (KHT) have been widely cultivated. These non-pigmented domestic rices are hard grain and odorless when compared to *Jasmine* (Thai *Hom Mali*) rice. Thus, local rice should be used as a raw material for further processed foods rather than just for normal household consumption. Recently, grain-based drinks have increased in demand [1]. Apart from the rice variety, the processing of rice in particular germination can affect the compositional, nutritional, and physicochemical properties. Germination is the metabolic process that increases the synthesis of secondary metabolites, e.g. phenolic compounds and γ-aminobutyric acid (GABA) [2]. During germination, the improvement of nutritional and organoleptic properties can be found due to the production of various nutrients (e.g. free amino acid (FAA), vitamin, and mineral) and the reduction in antinutrient levels (e.g. trypsin inhibitor, the release of flavoring compounds, and the softening of texture) [3]. The biochemical, nutritional, and sensory characteristics of germinated grains depend on the sprouting condition [3]. The germination conditions of indigenous Thai rice need to be optimized in order to maximize the enzymatic activities and nutritional qualities of the resulting germinated rice. To produce beverages from the germinated rice, mashing is a sequential process after sprouting in which saccharification and other enzymatic degradation reactions occur. During mashing, three basic biochemical processes typically take place, including cytolysis, proteolysis, and amylolysis which yield β-glucan, FAA, and sugar, respectively [4]. The enzymatic activities and the optimal mashing conditions may vary among the cereal origins. This study aimed to investigate the effects of the germination time on the α-amylase activity and nutritional values of germinated rice from two domestic Southern Thai rice cultivars, namely KHT and KMR. The appropriate germination time was selected for the sequential optimization of the mashing condition. The nutritional composition, antioxidant activities, and angiotensin I-converting enzyme (ACE) inhibitory activity of the germinated rice extract beverages were investigated in comparison with those made from *Jasmine* (commercial Thai *Hom Mali* rice) and *Sungyod* (KGTC82239-2) (local pigmented rice) extract.

## Material and methods

### Rice paddies and chemicals

Two domestic Southern Thai rices (12% moisture), namely KMR and KHT, were harvested from rice paddies in Pak Phanang, Nakhon Si Thammarat, Thailand, in 2018. The germination ability of the paddies (at least 90% germination) was tested before being used in the experiment. All chemicals used for analyses were purchased from Sigma-Aldrich Corp. (St. Louis, MO, USA).

### Effect of germination time on α-amylase activity and nutritional composition

From the preliminary study, soaking in mild acid water (pH 5) at 35°C in the dark for 48 h while changing the soaking water every 6 h was considered to be the best condition for the germination of the domestic Southern Thai rices due to nutritional and economical points of view. However, the effect of germination time was excluded from our previous study. This parameter plays an important role in both the enzymatic activity and nutritional quality of germinated rice. Hence, the effect of germination times (0–96 h) in mild acid water (pH 5) at 35°C in the dark on the α-amylase activity and nutritional composition of KMR and KHT was investigated. The pH of the soaking water was adjusted to 5 using 1 M of citric acid. The samples from different germination times were taken by cutting the root and grinding for analyses. The α-amylase activity was spectrophotometrically assayed following the method of Baker [5] and expressed as μmol glucose release/g/min. The total sugar, GABA, FAA, and thiamine contents were spectrophotometrically determined according to A.O.A.C [6], Zhang et al. [7], Benjakul and Morrissey [8], and Liu et al. [9], respectively.

### Effect of mashing condition on nutritional composition

The combined effect of the temperature and the pH was investigated based on the previous literatures for maximizing nutritional values of germinated rice during mashing for 30 min. The germinated rice were ground using an MK 5087M Panasonic Food Processor (Selangor Darul Ehsan, Malaysia). The ground sample was added with water (1:4, w/v), adjusted to the desired pH using 1M of citric acid, and incubated at desired temperature/pH (55°C/pH 4.5, 45°C/pH 5.3, 60°C/pH 5.5, and 55°C/pH 6.0) for 30 min with continuous stirring at 150 rpm using an IKA overhead stirrer equipped with a propeller (Model RW 20.n, Staufen, Germany). To obtain the final germinated rice extract, centrifugation was done at 5,000×g for 5 min (RC-5B plus centrifuge, Sorvall, Norwalk, CT, USA), and then the supernatant was pasteurized at 63°C for 30 min [10]. The analyses were done for proximate composition (including moisture (A.O.A.C method number 950.46), crude protein (A.O.A.C method number 928.08, Kjeldahl factor of 6.25), crude fat (A.O.A.C method number 963.15), and ash (A.O.A.C method number 920.153)) [6], total phenolic content (TPC), total flavonoid content (TFC), and phytic acid [11]. The TPC was determined using the Folin-Ciocalteu procedure and expressed as mg gallic acid equivalent (GAE)/mL. The TFC was analyzed using the aluminium chloride colorimetric method and expressed as μg rutin equivalent (RE)/mL. GABA, FAA, thiamine, and total sugar content were also measured using the same methods described above.

The germinated rice extracts prepared from the selected mashing condition were determined for their mineral composition (potassium, magnesium, calcium, manganese, iron, zinc, and copper) using an inductively coupled plasma optical emission spectrophotometer (ICP-OES) (Perkin-Elmer, Model 4300 DV, Norwalk, CT, USA) according to A.O.A.C [6].

### Antioxidative and ACE inhibitory activities of germinated rice extracts

The germinated rice extracts from KMR and KHT prepared by the optimum mashing condition were analyzed for *in vitro* antioxidative and ACE inhibitory activities compared to those from *Jasmine* rice and *Sungyod* rice at the same processing condition. DPPH^•^, ABTS^•+^, OH^•^, and H_2_O_2_ scavenging activities, the reducing power, and chelating activity were measured according to the method of Limsuwanmanee et al. [12]. The method of Cushman and Cheung [13] was used to determine the ACE inhibitory activity.

### Statistical analysis

A completely randomized design was used and the entire experiment was replicated three times. Data were subjected to analysis of variance (ANOVA). Comparison of means was carried out by Duncan’s multiple-range test to identify significant differences (p<0.05) among treatments. A *T*-test was used for pairwise comparison. Statistical analysis was done using the Statistical Package for the Social Sciences (SPSS) 10.0 for Windows (SPSS Inc., Chicago, IL, USA).

## Results and discussion

### Effect of germination time on α-amylase activity and total sugar content

**Fig 1:**
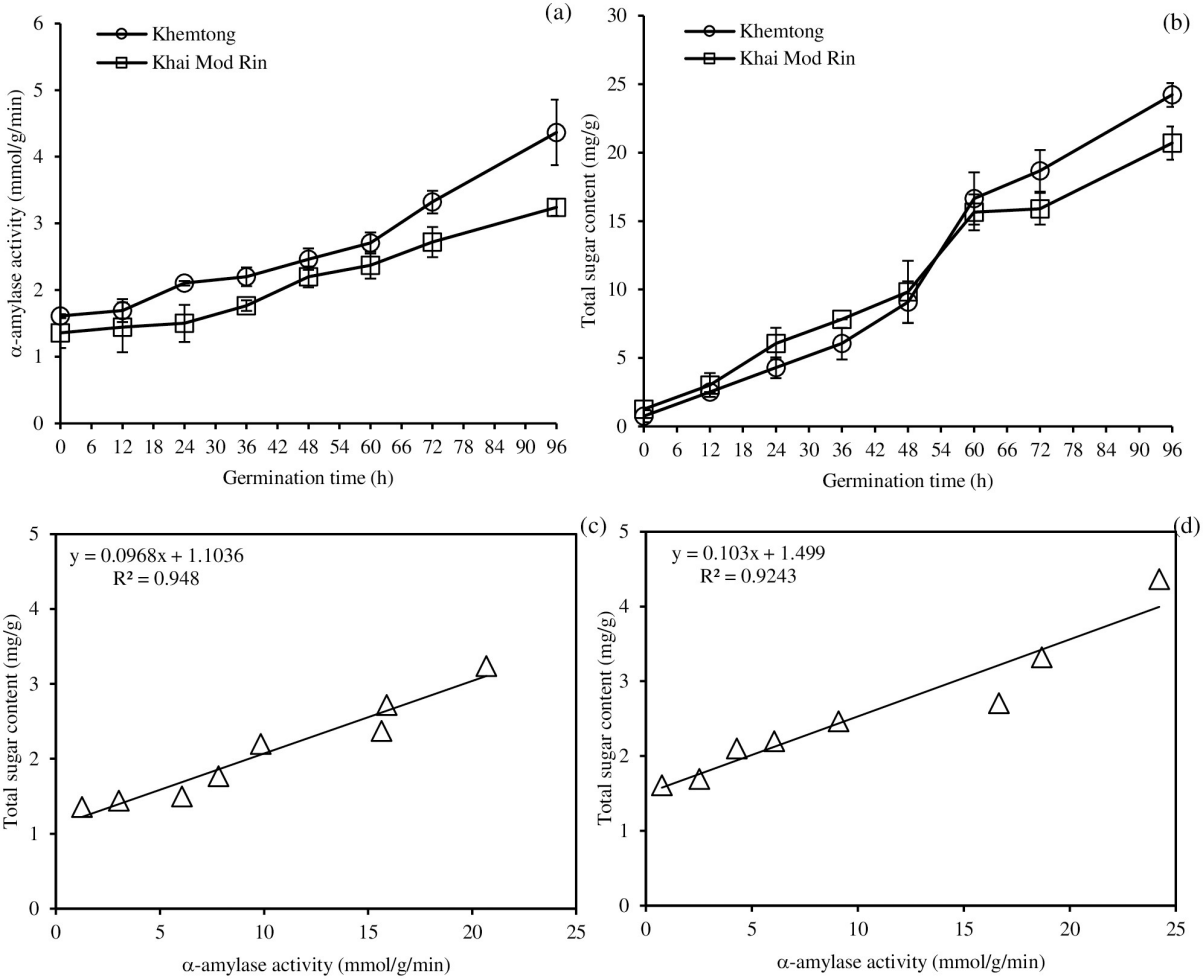
Effect of germination time on α-amylase activity (a), total sugar content (b) and their correlation curves (c-d) of two indigenous Southern Thai rices, *Khemtong* and *Khai Mod Rin*, germinated at 35°C in the dark. The bars indicate standard deviation from triplicate determinations.

The α-amylase activity of the germinated KHT and KMR as a function of germination time is shown in Fig 1a. The α-amylase activity was directly proportional to the germination time, reaching a maximum at 96 h. The KHT malt had a slightly higher α-amylase activity than the KMR one throughout the germination time, indicating a higher starch hydrolytic rate of the former. The enzyme production and the activity variation of rice were governed by the cultivar [14]. Principally, α-amylase, β-amylase, limit dextrinase, and α-glucosidase are important enzymes for malting, which hydrolyze the starch into simple sugars [15]. In this study, only α-amylase activity was measured because α-amylase activity has been used to monitor the progression of rice germination [16]. However, the total sugar content which is a product from carbohydrase activities was monitored during germination.

An increasing trend of the total sugar content of the germinated KHT and KMR is depicted in Fig 1b. A higher total sugar content was found in the germinated KHT, demonstrating a greater sugar production rate during germination. This was highly correlated with the α-amylase activity, where starch hydrolysis led to sugar formation. During seed germination, amylolytic enzymes were activated and facilitated the release of soluble starch and reducing sugars. Maltose was the most predominant sugar in germinated legumes (~74–80% of total sugar) [17].

The positive correlations between the amylase activity and the total sugar content of KMR and KHT rice during germination are presented in Fig 1c and 1d, respectively. Linear correlations between the sugar formation and the amylase activity were found in both rice cultivars with an R^2^ of 0.9480 and 0.9234 for KMR (Fig 1c) and KHT malts (Fig 1d), respectively. From the regression line, the germinated KHT tended to have a higher slope than the germinated KMR, suggesting a faster rate of starch hydrolysis of the former.

### Effect of germination time on GABA, FAA, and thiamine contents

**Fig 2:**
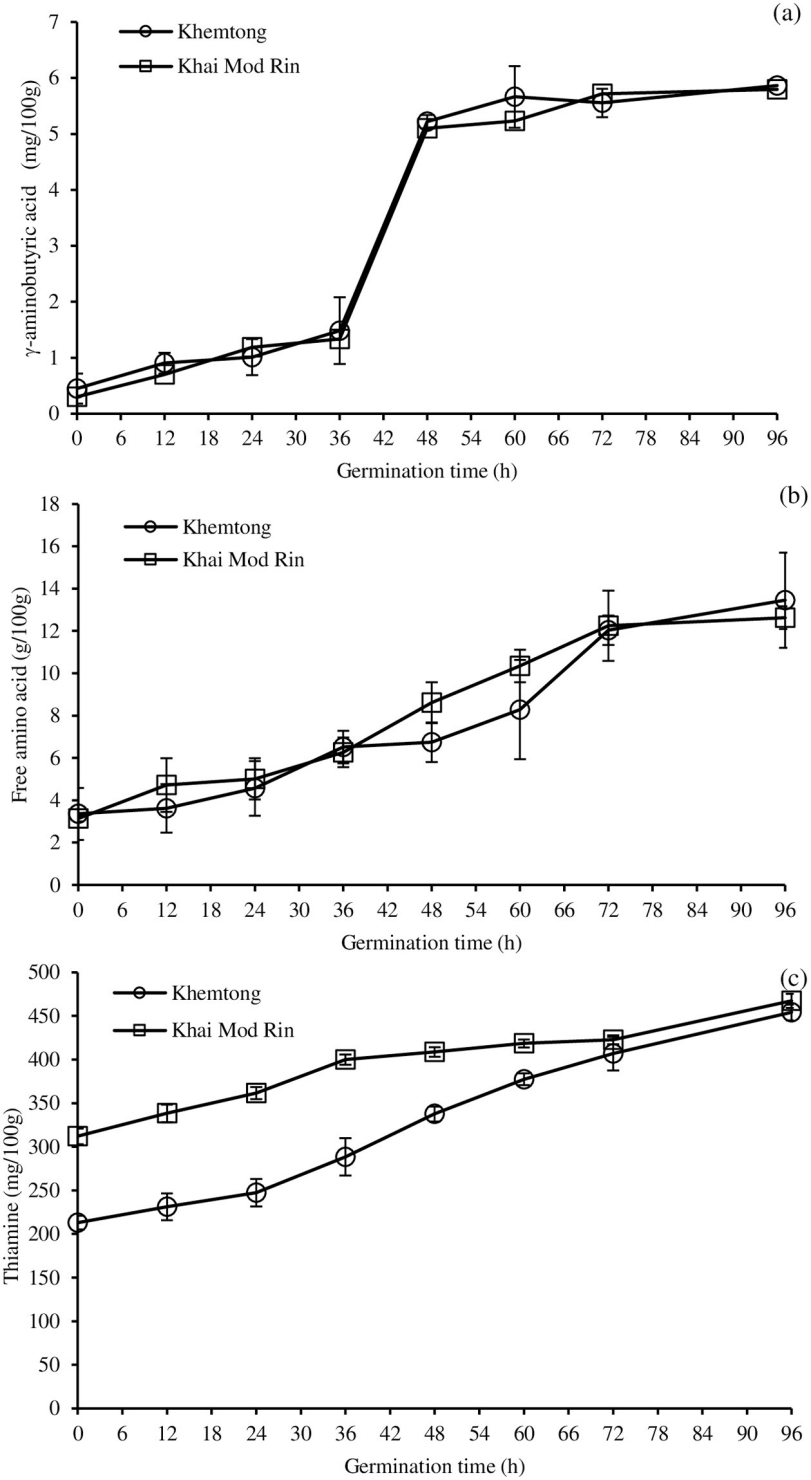
Effect of germination time on γ-aminobutyric acid (GABA) content (a), free amino acid content (b), and thiamine content (c) of two indigenous Southern Thai rices, *Khemtong* and *Khai Mod Rin*, germinated at 35°C in the dark. The bars indicate standard deviation from triplicate determinations.

No significant difference in the GABA content for both germinated rices was observed throughout the germination period (p>0.05, Fig 2a), indicating a comparable GABA formation rate. The GABA content for both germinated rices slightly increased during the first 36 h, markedly increased up to 48 h, tended to insubstantially increase up to 72 h, and remained constant until the end of germination (Fig 2a). This was probably due to the different activity of the glutamate decarboxylase (GAD) during germination. Moreover, the release of free glutamic acid, a substrate of GABA synthesis, might have influenced the GABA formation. The maximum GABA content of both paddies was achieved after 72 h of germination. A sharp increase in the GABA content between 36 to 48 h was observed. This might be due to the optimal condition for the reaction between the L-glutamic acid and GAD. At this period, free L-glutamic acid might be released to the greatest extent due to the destruction of the cellular structure.

An increasing trend in the FAA content was observed in both germinated rices throughout the germination period (Fig 2b). At the same period, no significant difference in the FAA content was detected among the samples (p>0.05), indicating the same protease activity of both rices during germination. However, the different pattern of changes in FAA and GABA were noticeable, indicating the different activities of enzymes responsible for the proteolysis and decarboxylation of L-glutamic acid. The maximum FAA content of both germinated rices can be achieved after 72–96 h of germination (Fig 2b). Typically, the FAAs are synthesized by the hydrolysis of the storage proteins in the grain endosperm by the endogenous proteinases [18]. The FAAs and peptides can be formed during germination which can nourish the resulting germinated rice.

The time-course development of thiamine during the germination of the KHT and KMR rice is depicted in Fig 2c. The levels of the thiamine in both cultivars increased during germination. The initial thiamine content of the germinated KMR was higher than that of the KHT one, suggesting a different thiamine biosynthesis upon soaking. Up to 60 h of the germination, the thiamine content of the germinated KMR was still greater than the KHT. Then, the levels went upward, reaching comparable values at 72–96 h (Fig 2c), suggesting a greater thiamine formation rate of the germinated KHT at the final stage of the germination. The germination conditions (i.e. the number of rinses, light levels, and seed germination time) play an important role on the vitamin contents [19].

Overall, the optimum germination time for both rices was 96 h, yielding the highest contents of GABA, thiamine, free amino acid, total sugar, and α-amylase activity (p<0.05). Then, the 96-h germinated rices were selected for the sequential optimization of mashing conditions.

### Effect of mashing condition on nutritional composition of germinated rice extracts

To prepare the germinated rice extracts, the mashing of the two germinated rices was carried out, where saccharification and flavor development proceeded. Mashing includes a complex system of chemical and biochemical reactions. Hydration of starch granules, enzymatic hydrolysis of starch, and proteolysis generally take place, yielding soluble sugars, various nutrients, and flavor substances.

### Proximate composition

The effects of the mashing conditions on the proximate composition of the germinated KHT and KMR extracts are shown in Table 1. The highest total protein content of the germinated KMR extract was found when the mashing was done at 60°C/pH 5.5 (p<0.05), followed by at 55°C/pH 6.0, 45°C/pH 5.3, and 55°C/pH 4.5. Under this condition, the highest degree of rice matrix degradation and proteolysis could occur, resulting in increased extractable protein, soluble peptide, and FAA contents. A similar trend was also found in the germinated KHT extract. Mohan et al. [17] reported that proteins were hydrolyzed during brown rice germination. Negligible lipid content was observed in both germinated rice extracts, ranging from 0.01 to 0.14% (Table 1). This may relate to the low content of lipid in the initial rice together with the extensive lipolysis by endogenous lipase during seed germination and mashing. The fiber content of both germinated rice extracts was in the range of 1.70–2.65%. The germinated KHT extract had a higher fiber content than the KMR for all the mashing conditions (p<0.05), indicating a higher dissolvable fiber content in the germinated KHT extract. Since crude fiber was determined in the aqueous extract of the germinated rice, it was soluble fiber. This soluble fiber was a part of the dietary fiber because dietary fiber has two main components, including soluble fiber (e.g. β-glucan) and insoluble fiber (e.g. cellulose and lignin) [20]. During mashing, β-glucan can be produced due to cytolysis [4]. The ash content of both germinated rice extracts was relatively low, ranging from 0.15 to 0.33%. Ash refers to the mineral composed in the germinated rice extracts. Thus, the ash content can vary depending on the rice cultivar and the solubility or the extractability of the individual mineral in the aqueous extract.

**Table 1:** The nutritional composition of germinated rice extracts from two local Southern Thai rices that underwent different mashing conditions.

Parameter	Mashing condition			
	45°C/pH 5.3	55°C/pH 4.5	55°C/pH 6.0	60°C/pH 5.5
*Khai Mod Rin* malt extract				
Proximate composition (dry basis)				
•Protein content (%)	3.33±0.02b	3.10±0.17a	3.48±0.03c	4.20±0.21d
•Fat content (%)	0.07±0.03c	0.04±0.02b	0.01±0.01a	0.01±0.00a
•Fiber content (%)	1.87±0.28a	1.70±0.19a	1.81±0.08a	1.82±0.28a
•Ash content (%)	0.20±0.01a	0.33±0.04b	0.27±0.08b	0.32±0.00b
Antioxidant compounds				
•Total phenolic content (mg GAE/mL)	3.10±0.04a	3.75±0.02b	3.96±0.06c	4.04±0.02d
•Total flavonoid content (μg RE/mL)	48.72±1.03c	42.81±0.42a	45.33±1.21b	51.68±0.20d
•Phytic acid content (μg/L)	47.23±0.26a	46.26±0.80a	47.47±0.59a	47.16±0.85a
GABA (mg/L)	12.03±0.54b	11.62±0.55b	8.67±0.54a	19.29±0.54c
Free amino acid (mg/L)	0.13±0.02a	0.14±0.01a	0.14±0.01a	0.14±0.01a
Thiamine (μg/L)	0.35±0.02b	0.027±0.01a	0.79±0.01c	1.00±0.02d
Total sugar content (mg/L)	231±1c	173±1a	211±1b	246±0d
*Khemtong* malt extract				
Proximate composition (dry basis)				
•Protein content (%)	4.93±0.04b	4.48±0.05a	4.88±0.04b	5.02± 0.08c
•Fat content (%)	0.14±0.08d	0.03±0.01b	0.01±0.01a	0.07±0.02c
•Fiber content (%)	2.03±0.19a	2.15±0.62a	2.65±0.61a	1.99±0.06a
•Ash content (%)	0.33±0.02d	0.15±0.02a	0.24±0.01b	0.26±0.00c
Antioxidant compounds				
•Total phenolic content (mg GAE/mL)	3.27±0.01a	3.49±0.04b	3.96±0.05c	4.06±0.05d
•Total flavonoid content (μg RE/mL)	52.21±0.90c	33.55±0.65a	44.15±0.80b	59.30±0.53d
•Phytic acid content (μg/L)	41.04±1.12a	41.80±0.40a	40.24±0.45a	40.56±0.84a
GABA (mg/L)	10.37±0.36b	5.55±0.41a	10.05±0.55b	11.92±0.56c
Free amino acid (mg/L)	0.18±0.01a	0.18±0.01a	0.19±0.02a	0.19±0.01a
Thiamine (μg/L)	0.20±0.01b	0.02±0.00a	0.30±0.01c	0.86±0.01d
Total sugar content (mg/L)	294±1b	310±1c	221±1a	337±0d

*Values are given as means ± SD from triplicate determinations.

**Different letters in the same row indicate significant differences (p < 0.05).

GAE = gallic acid equivalent, RE = rutin equivalent.

### TPC, TFC, and phytic acid content

The TPC, TFC, and phytic acid contents of the germinated KHT and KMR extracts prepared by the different mashing conditions are shown in Table 1. The highest TPC of both extracts was obtained when mashing was done at 60°C/pH 5.5, followed by at 55°C/pH 6.0, 55°C/pH 4.5, and 45°C/pH 5.3 (p<0.05). Carvalho et al. [21] stated that the structural changes during germination and mashing involved many enzyme degradations, which can facilitate the extraction of free polyphenols. The enzyme activities may differ considerably depending on temperature and pH conditions. Generally, the identified phenolic acids in the endosperm, bran, and whole grain of rice are ferulic acid, p-coumaric acid, sinapic acid, gallic acid, protocatechuic acid, p-hydroxybenzoic acid, vanillic acid, and syringic acid [22]. An individual phenolic may dissolve in an aqueous medium differently, which can influence the polyphenol concentration and the antioxidant activity of the final extract. At the same mashing condition, the KHT extract had a higher TPC than the KMR. This might be due to the different content of original phenolic among the rice varieties. The TFC of the germinated rice extracts are given in Table 1. Mashing at 60°C/pH 5.5 showed the greatest TFC in both extracts (p<0.05). The enzymatic cellular disruption during mashing led to the release of the flavonoids from the intact malt cell. Rice bran is rich in phenolics and flavonoids compared to other parts [22]. A leaching of flavonoids from the ground germinated rice (with rice hull) led to the accumulation of flavonoids in the extract. No significant differences in the phytic acid content prepared by different mashing conditions were noted in both extracts (p>0.05) (Table 1). At the same mashing condition, the germinated KMR extract seemed to have a higher phytic acid content than the KHT (Table 1). A reduction of phytic acid, an anti-nutrient compound, has been reported during sprouting which is likely to be due to the indigenous phytase activity in germinated seeds [23].

### Nutrients

The effects of the mashing conditions on GABA, FAA, thiamin, and total sugar contents of both germinated rice extracts are shown in Table 1. Both the germinated KHT and KMR extracts prepared at 60°C/pH 5.5 showed the highest GABA concentration (p<0.05). This condition could stimulate the GAD to catalyze the GABA production from L-glutamic acid. The GABA accumulation of the rice germ is dependent on the GAD activity and the substrate concentration [24]. The instability of the GABA in some mashing conditions may influence its content in the final extract. The germinated KMR extract showed a significantly higher accumulated GABA content than the KHT at the same mashing condition (Table 1).

No significant differences in the FAA content of both extracts were found among the mashing conditions (p>0.05) (Table 1). The final level of the FAA in the extract can be affected by both the formation rate (via the proteolysis of malt proteins) and the transformation rate (via the decarboxylation to GABA or the Maillard reaction). The higher the protein hydrolysis rate the greater the remaining FAA content. At the same mashing condition, the germinated KMR extract (0.13–0.14 mg/mL) had a slightly lower FAA content than the KHT extract (0.18–0.19 mg/mL). These values were rather low compared to the desired FAA concentration of 2–2.5 mg/mL after mashing [4]. This supported the higher GABA level in the germinated KMR extract. The released L-glutamic acid might be transformed to the GABA during germination and mashing, demonstrating a higher GABA content with a lower remaining FAA in the germinated KMR extract (Table 1). Kühbeck et al. [4] indicated that the formation of the FAA, due to endoproteolytic activity, during mashing was considerably smaller than during seed germination.

The thiamine content of both germinated rice extracts was different, depending on the mashing condition (Table 1). The greatest thiamine concentration in both extracts was observed when they were mashed at 60°C/pH 5.5, followed by 55°C/pH 6.0, 45°C/pH 5.3, and 55°C/pH 4.5 (p<0.05), suggesting the important role of temperature and pH during mashing on the thiamine content. The lowest thiamine content was found in the extracts mashed at pH 4.5, which was the lowest pH employed. Windheuser and Higuchi [25] reported on the instability of thiamine under acidic conditions. The germinated KMR extract exhibited a slightly greater thiamine concentration than the KHT. This was due to the higher thiamine content in the sprouted KMR (Fig 2c), resulting in a greater level of thiamine in the finished extract.

The total sugar content of the germinated KMR and KHT extracts was largely influenced by the mashing conditions (Table 1). The sugar content of the germinated KMR extracts ranged from 172.90 to 246.46 mg/mL, whereas the germinated KHT extract showed a higher sugar content (220.74–336.55 mg/mL). Since the mashing period was fixed at 30 min, a lower sugar concentration of the final extract was clearly related to the carbohydrases activities. For example, a higher α-amylase activity of the KHT extract was found during germination (Fig 1a). This α-amylase might also continuously be active during mashing, leading to a higher sugar content of the final product (Table 1). In terms of the sugar production, the KHT rice was more suitable for the germinated rice extract production than the KMR. Regarding the mashing condition of both extracts, mashing at 60°C/pH 5.5 rendered the extract with the greatest sugar content while the lowest was found at 55°C/pH 6.0 (p < 0.05). Owuama [26] suggested that the optimum condition for α-amylase and β-amylase was at 56°C/pH 4.0 and 70°C/ pH 5.5, respectively. However, the optimum temperature and pH were dependent on the source of enzymes.

The mineral composition of the germinated KMR and KHT extracts obtained from the optimum mashing condition (60°C/pH 5.5) is shown in Table 2. Potassium was the most abundant mineral observed in both samples, followed by magnesium, and calcium. The minor minerals were found in the order of manganese > iron > zinc > copper. No significant differences in calcium, zinc, and copper were detected among the extracts (p>0.05). However, significantly greater magnesium, potassium, manganese, and iron were noticed in the germinated KMR extract compared to the KHT (Table 2). The composition and amount of minerals in the extracts are largely involved in their parent rice. Compared to the other malt extracts, the germinated KMR and KHT extracts had higher contents of iron, zinc, copper, and manganese than the Nigerian malt extracts [27]. In comparison with some fruit juices, the germinated KMR and KHT extracts contained a higher content of magnesium than apple juice, grapefruit juice, and lemon juice, but they had a comparable content of magnesium to orange juice and grape juice [28]. However, the potassium and calcium contents in the germinated KMR and KHT extracts were lower than those found in some beverages as reported by Buglass [28]. Moreover, the iron and zinc contents in the germinated KMR and KHT extracts were quite similar to various commercial beverages [28]. The differences probably originate from the endogenous mineral content of the raw material, the processing, and the mineral fortification.

**Table 2:** Mineral composition of germinated rice extracts made from two domestic Southern Thai rices.

Mineral	Germinated *Khai Mod Rin* extract (mg/L)	Germinated *Khemtong* extract (mg/L)
Potassium (K)	274±2b	247±3a
Magnesium (Mg)	110±1b	107±1a
Calcium (Ca)	11.2±0.4a	10.4±0.1a
Manganese (Mn)	1.4±0b	1.3±0a
Iron (Fe)	0.9±0b	0.5±0a
Zinc (Zn)	0.3±0a	0.3±0a
Copper (Cu)	0.1±0a	0.1±0a

*Values are given as means ± SD from triplicate determinations.

**Different letters in the same row indicate significant differences (p < 0.05).

### Antioxidative and ACE inhibitory activities of germinated rice extracts

**Fig 3:**
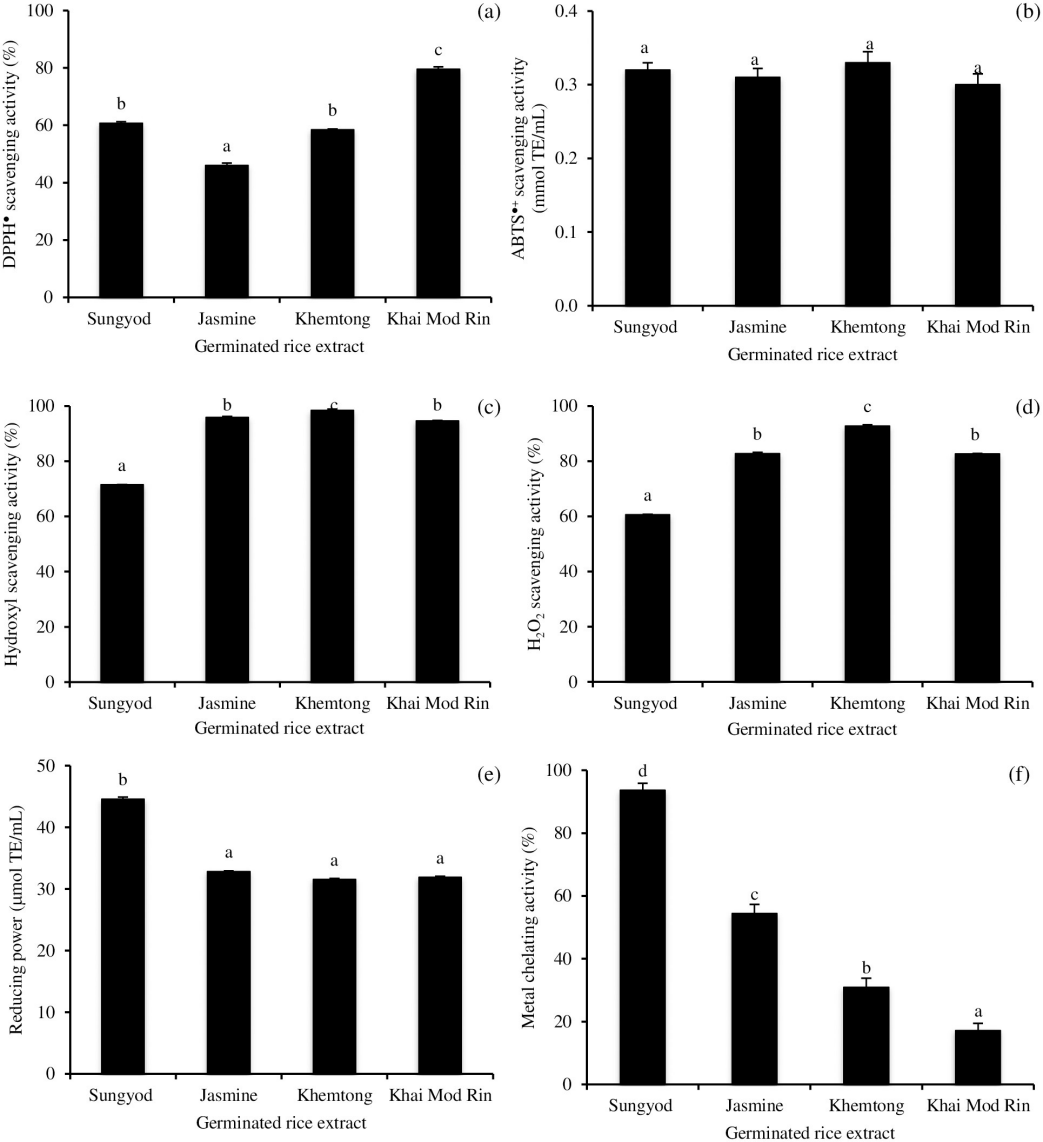
Effect of mashing condition on DPPH radical scavenging activity (a), ABTS radical scavenging activity (b), hydroxyl radical scavenging activity (c), hydrogen peroxide scavenging activity (d), reducing capacity (e), and metal chelating ability (f) of germinated rice extracts made from two indigenous Southern Thai rices, *Khemtong* and *Khai Mod Rin*, in comparison with germinated *Jasmine* and *Sungyod* extracts. The bars indicate standard deviation from triplicate determinations. Different letters on the bars indicate significant differences (p<0.05).

The individual or synergist effect of several compounds in the germinated rice extracts such as polyphenols, flavonoids, anthocyanin, phytic acid, vitamins, Maillard reaction products, peptides, and other phytonutrients may participate in the antioxidant and ACE inhibitory activities. The radicals scavenging activities against the DPPH^●^, ABTS^●+^, and OH^●^ of the germinated KMR and KHT extracts in comparison with the germinated *Sungyod* and *Jasmine* extracts are shown in Fig 3a to 3c. The highest DPPH^●^ scavenging activity was observed in the germinated KMR extract, followed by the germinated KHT/ *Sungyod* and *Jasmine* extracts (p<0.05; Fig 3a). No significant difference in the ABTS^●+^ scavenging activity was noticed among all the extracts (p>0.05; Fig 3b). Interestingly, the extracts from all three non-pigmented rices showed a higher OH^●^ scavenging activity than the germinated *Sungyod* pigmented rice extract (p<0.05; Fig 3c), which contained the highest phenolic content (4.84 mg/mL). The germinated KHT extract possessed the highest H_2_O_2_ scavenging activity, followed by the extracts from the germinated KMR/*Jasmine* and *Sungyod* extracts (Fig 3d). The radical scavenging capacity of the anthocyanin, a major phenolic compound found in the red-purple *Sungyod* rice, has been largely reported. However, the activity was similar or slightly lower than the other phenolic compounds. Kähkönen and Heinonen [29] indicated that anthocyanins and anthocyanidins possessed lower DPPH^●^ scavenging activity than gallic acid, chlorogenic acid, and rutin. Different forms of anthocyanin may act differently in the radical scavenging activity [30]. Pereira et al. [30] reported that the radical scavenging activity of anthocyanin is dependent on the degree and position of hydroxylation and methoxylation in the B ring. From the structure-activity relationship, the hydroxylation at C3′ and C5′ improved the H-donating capacity and thus the B-ring was primarily involved in the electron donation [29]. From the results, the scavenging activities against free radicals and reactive oxygen species (ROS) of the germinated rice extract were governed by the rice cultivar.

The reducing abilities of the germinated rice extracts are depicted in Fig 3e. The germinated *Sungyod* extract with the highest TPC (4.84 mg/mL) showed the highest reducing capacity (p<0.05). No significant difference in the reducing power was detected in the extracts from all three non-pigmented rices (p>0.05), which contained similar TPC (3.79–3.95 mg/mL). Solgajová et al. [31] reported that the reducing capacities of the malt beverages enriched with hop or various amounts and types of bee pollen were influenced by the TPC. Typically, the reducing properties are associated with the presence of reductones, which can be found in the germinated rice extracts to some extent due to the possible Maillard reaction. However, phenolic compounds are reported to act in a similar fashion as reductones by donating electrons and reacting with free radicals to convert them to more stable products and terminating the free radical chain reaction [32].

Ferrous iron can promote the generation of ROS, such as superoxide anion, hydroxyl radical, and non-free radical species, and accelerate lipid peroxidation by the Fenton reaction [12]. The ferrous chelating activities of the germinated rice extracts are given in Fig 3f. The greatest metal chelating capacity was found in the germinated *Sungyod* extract (p<0.05), followed by the *Jasmine*, KHT, and KMR extracts. The extract from pigmented rice possessed superior metal chelation than those from non-pigmented extracts. The metal chelating activity was directly proportional to the polyphenol concentration in the germinated rice extract. The extracts derived from the germinated KHT and KMR had powerful antioxidant properties against various antioxidant systems. Therefore, the daily consumption of beverages made from these germinated Southern Thai indigenous rices may help retard and/or prevent oxidative stress in the human body.

**Fig 4:**
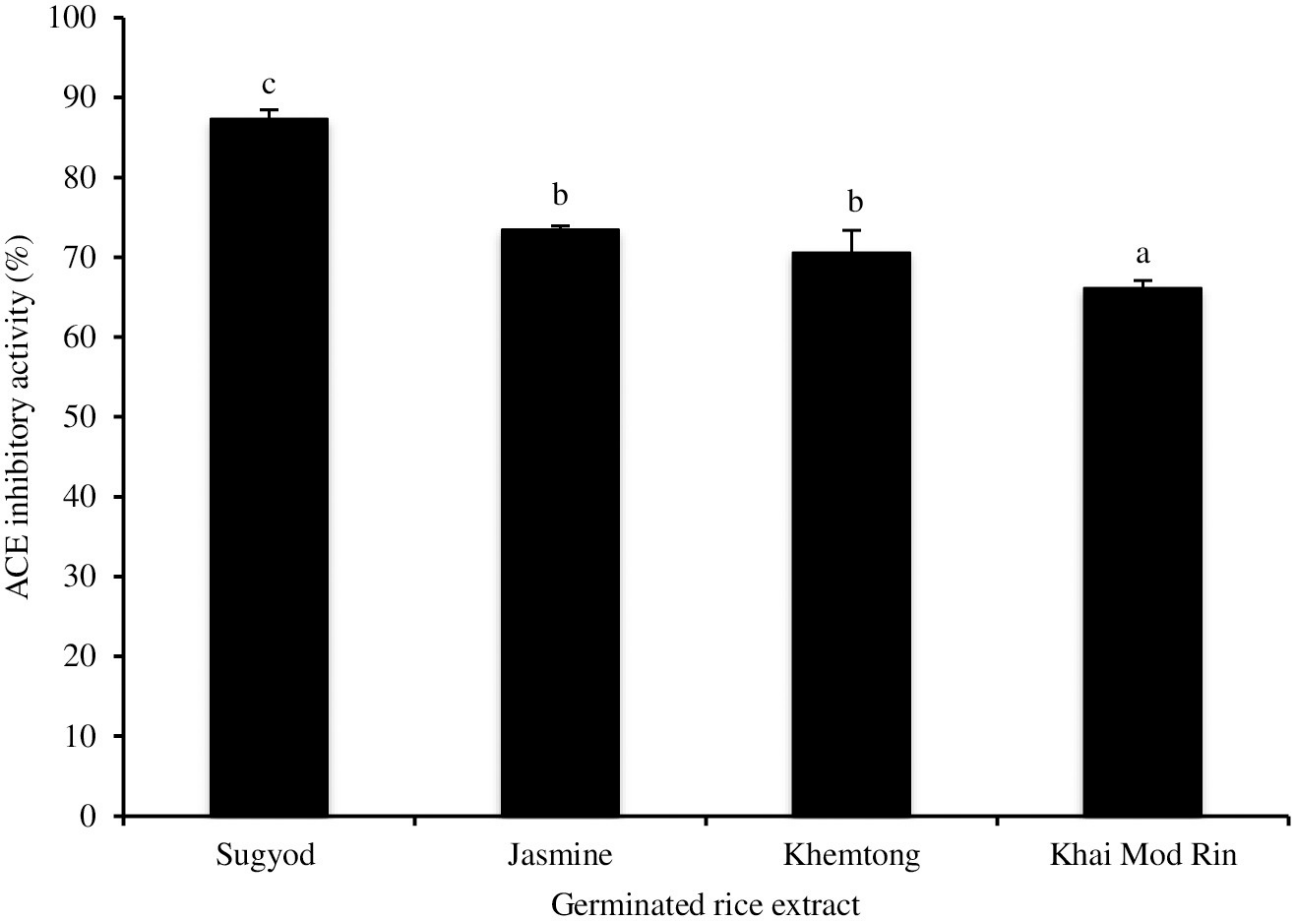
Effect of mashing condition on angiotensin I-converting enzyme (ACE) inhibiting activity of germinated rice extracts made from two indigenous Southern Thai rices, *Khemtong* and *Khai Mod Rin*, in comparison with germinated *Jasmine* and *Sungyod* extracts. The bars indicate standard deviation from triplicate determinations. Different letters on the bars indicate significant differences (p<0.05).

The ACE inhibitory activities of the germinated KMR and the KHT extracts compared to the germinated *Jasmine* and the pigmented *Sungyod* extracts were measured (Fig 4). Significant differences in the ACE inhibitory activities (p<0.05) were noted among the extracts made from the different rice varieties. The highest ACE inhibitory activity was noticed in the extract of germinated *Sungyod*, followed by the *Jasmine*/KHT and KMR extracts. The difference might be caused by the varying types and concentrations of the anti-ACE agents, (e.g. polyphenols, GABA, peptides, vitamins, etc.) presented in the tested extracts. The peptides generated during rice germination and mashing may contribute to the ACE inhibiting property. GABA was also reported as a potential substance to reduce blood pressure in hypertensive subjects [33]. It should be noted that all extracts made from the germinated non-colored rice had lower ACE inhibitory activities than the pigmented rice extract. The ACE inactivation potential of anthocyanins has been reported [34].

**Fig 5:**
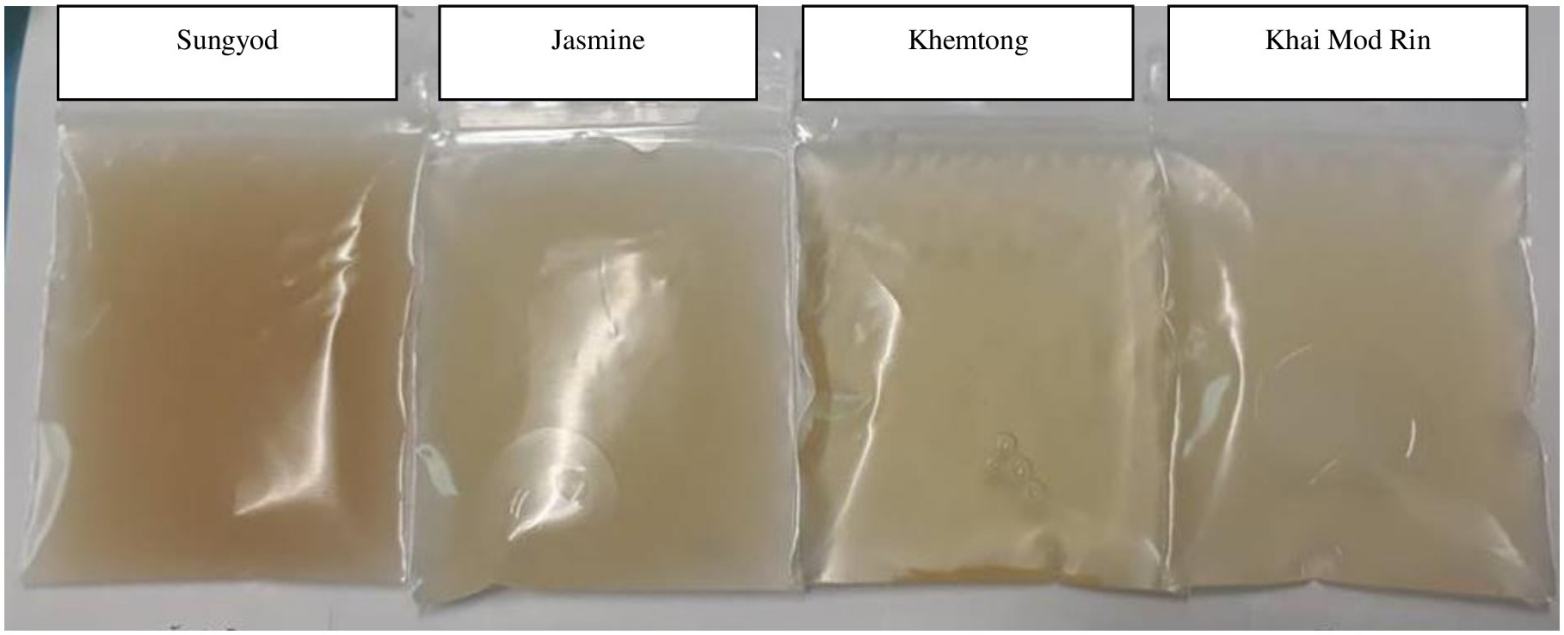
The appearances of the final germinated rice extracts prepared from different rice cultivars.

The appearances of the final germinated rice extracts prepared from the different rice cultivars including *Sungyod*, *Jasmine*, KHT and KMR are shown in Fig 5.

## Conclusion

Germination time influenced the α-amylase activity and the compositional contents of the germinated indigenous Southern Thai rices. The correlation between the α-amylase activity and the total sugar content was observed. The germinated KHT possessed a higher α-amylase activity than the KMR during sprouting. The mashing condition markedly affected the nutritional contents of the resulting germinated rice extracts. The extracts mashed at 60°C/pH 5.5 showed the greatest overall nutrients with a high total sugar concentration. Both germinated rice extracts had superior anti-radicals ability compared to the germinated *Sungyod* extract but slightly lowered reducing power, chelating activity, and ACE inhibitory capacity. As a consequence, both local Thai rice cultivars can be used as alternative raw materials for the production of germinated rice extracts. The processes of germination and mashing for the extract production are practical and achievable, which can be promptly transferred to community enterprises in Thailand.
